# Case Report: When catatonia-like symptoms are not catatonia: Guillain–Barré syndrome in a patient with schizophrenia

**DOI:** 10.3389/fpsyt.2026.1826383

**Published:** 2026-04-21

**Authors:** Lishan Ren, Wenjuan Liu, Hongjing Mao, Kaiyuan Zhang

**Affiliations:** Department of Sleep Medicine, Affiliated Mental Health Center & Hangzhou Seventh People’s Hospital, Zhejiang University School of Medicine, Hangzhou, China

**Keywords:** case report, catatonia, Guillain-Barré syndrome, lumbar puncture, schizophrenia

## Abstract

**Introduction:**

Schizophrenia is a severe psychiatric disorder. Catatonia is relatively common in schizophrenia; its main manifestations include catatonic stupor, mutism, negativism, and other psychomotor symptom clusters. Patients with schizophrenia are at increased risk of infections, which are also recognized triggers for Guillain–Barré syndrome (GBS). GBS typically presents with limb weakness, paresthesia, facial weakness, respiratory muscle paralysis, and autonomic symptoms, and may similarly result in severe immobility and impaired communication. Consequently, when schizophrenia (especially with catatonic features) coexists with GBS, history taking and clinical assessment can be challenging, increasing the risk of misdiagnosis or delayed diagnosis.

**Case presentation:**

We report a 28-year-old man with a 3-year history of schizophrenia who was found after 2 weeks of lost contact and admitted emergently. His first episode featured hallucinations, persecutory delusions, and catatonia, which remitted with antipsychotic and other medications treatment. He subsequently relapsed twice after discontinuing medication; both relapses presented mainly with hallucinations and delusions without catatonia and remitted after re-treatment, followed by regular risperidone maintenance. In the current episode, hallucinations and delusions recurred and progressed to apathy, reduced speech and activity, and eventually complete mutism, immobility, and inability to perform self-care. Schizophrenia was initially diagnosed per DSM-5 criteria and risperidone was initiated. Further evaluation revealed pulmonary infection with limb weakness and decreased tendon reflexes, differing from the increased muscle tone typical of catatonia. Cerebrospinal fluid showed albuminocytologic dissociation (protein 0.61 g/L; leukocytes 1.2×10^6/L), and comorbid Guillain–Barré syndrome was considered. He was transferred to neurology and treated with intravenous immunoglobulin (25 g/day for 5 days) plus rehabilitation, with gradual improvement. During the 1-year follow-up, the patient continued risperidone 4 mg as maintenance to prevent psychiatric relapse; his symptoms had essentially resolved, and he returned to normal work and daily life.

**Conclusion:**

In psychiatric practice, mutism and immobility are often attributed to primary psychiatric illness, particularly in patients with established diagnoses, which can lead to missed or delayed detection of medical conditions. This case underscores the importance of thorough neurologic examination and timely investigations in uncooperative patients, especially after antecedent infection, including consideration of lumbar puncture to evaluate for comorbid neurologic disorders such as Guillain–Barré syndrome.

## 1 Introduction

Schizophrenia is a chronic, relapsing psychiatric disorder characterized primarily by impaired coordination of cognition, thinking, affect, and volition/behavior (1), and catatonia is also relatively common in schizophrenia (2). Earlier editions of the International Classification of Diseases (ICD) and the Diagnostic and Statistical Manual of Mental Disorders (DSM) included a “catatonic schizophrenia” subtype (3), but this classification was removed in ICD-11 and DSM-5 because of substantial heterogeneity across schizophrenia subtypes and their limited utility for treatment and prognosis (4–6). Accordingly, catatonia is now more commonly conceptualized as an independent syndrome or a specifier, underscoring the importance of thorough differential diagnosis and careful consideration of potential medical causes when catatonic features are encountered.

Catatonia is a severe neuropsychiatric syndrome occurring in approximately 2.3% to 9% of patients with psychiatric disorders (including autism spectrum disorder, mood disorders, and schizophrenia) (7). Its clinical presentation is dominated by psychomotor disturbances, encompassing symptom clusters such as negativism, catatonic stupor, mutism, echopraxia, and agitation (8). Crucially, catatonic-like states are not exclusive to primary psychiatric illnesses; they can also be precipitated by organic etiologies, such as central nervous system infections, focal brain injuries, and substance intoxication (9). Similarly, severe Guillain-Barré syndrome (GBS) compromising motor and respiratory muscles can manifest as profound paralysis and anarthria (10), closely mimicking the mutism and immobility characteristic of catatonia.

The phenotypic overlap between severe neurological deficits and catatonia creates a formidable diagnostic dilemma. When patients with a pre-existing psychiatric history present with such symptoms, families often seek psychiatric care initially. Consequently, clinicians are highly susceptible to diagnostic overshadowing—prematurely attributing the acute presentation to a psychiatric relapse while overlooking underlying, potentially life-threatening somatic illnesses (11). This diagnostic bias is exacerbated by the patient’s profound inability to communicate and the frequent lack of reliable informants, yielding incomplete medical histories that further prolong the definitive diagnosis (12). Such diagnostic delays can lead to inappropriate psychiatric interventions and adverse prognostic outcomes (13). To illustrate this critical clinical challenge, we report a case of a patient with schizophrenia whose concurrent Guillain-Barré syndrome closely mimicked a psychotic relapse. Hereby, we urge psychiatrists to prioritize comprehensive somatic and neurological examinations when evaluating patients presenting with acute mutism and immobility, thereby mitigating the risk of misdiagnosis and ensuring timely multidisciplinary intervention.

## 2 Case presentation

A 28-year-old unmarried man with a junior college education was first admitted to our hospital on July 10, 2024, for “recurrent auditory hallucinations, persecutory delusions, and psychomotor inhibition for 3 years, with acute worsening for 1 week”.

Psychiatric History: Three years earlier, without an obvious trigger, the patient developed auditory hallucinations (derogatory and running-commentary voices) and persecutory delusions. The condition rapidly progressed to mutism, immobility, and refusal to eat. He was diagnosed with schizophrenia at a local hospital and treated with aripiprazole 20 mg/day and other medications (the specific details of which the family could not recall), after which his symptoms remitted and he returned to normal functioning. Over the next 2 years, he had two psychotic relapses after discontinuing medication on his own. Both relapses were dominated by hallucinations and delusions, without psychomotor inhibition. During the past year, he took risperidone 2 mg/day as maintenance therapy, and his condition remained largely stable.

History of Present Illness: One month prior to admission, the patient traveled to Hangzhou for employment and suspectedly discontinued his medication. Two weeks prior to admission, he lost contact with his family. Three days prior, family members located him in a chaotic living environment; he was bedridden and exhibited disorganized speech, hallucinations, extreme weakness, and a low voice. Two days prior to admission, his condition deteriorated further to complete mutism, immobility, refusal of food and water, and inability to perform self-care (including toileting). He was admitted via emergency with a diagnosis of schizophrenia.

Personal and Family History: The patient had no significant past medical history. His premorbid personality was introverted and stubborn, with limited social interaction. Family history of psychiatric illness was negative.

Physical Examination: On admission, vital signs revealed a temperature of 38.6 °C, heart rate of 112 bpm, respiratory rate of 18 breaths/min, and blood pressure of 122/76 mmHg. The patient was conscious. Pupils were equal, round, and reactive to light. Chest auscultation revealed coarse breath sounds with bilateral lower lobe moist rales. Cardiac examination was normal. Abdominal percussion indicated a distended bladder (dullness three fingerbreadths above the pubic symphysis). Neurological examination revealed flaccid limbs; although formal assessment of muscle strength and tone was limited by poor cooperation, marked hypotonia was noted. Meningeal irritation signs and Babinski reflexes were negative.

Mental Status Examination (MSE): The patient was conscious but completely mute, lacking any non-verbal communication, rendering thought content inaccessible. He exhibited a blank stare, flat affect, profound avolition, and was entirely bedbound. He demonstrated passive compliance to limb manipulation (waxy flexibility) and lacked insight into his condition.

Ancillary tests: Laboratory tests suggested infection: white blood cell count 14.1×10^9/L, neutrophil count 12.16×10^9/L, and high-sensitivity C-reactive protein 16.1 mg/L. Blood biochemistry, thyroid function, sex hormone levels, and infectious disease screening (hepatitis B, syphilis, and HIV) were unremarkable, and the serum risperidone level was undetectable (0 ng/mL). Chest CT showed small infectious lesions in both lower lungs. Electrocardiogram, brain magnetic resonance imaging (MRI), and electroencephalogram (EEG) were within normal limits.

## 3 Diagnostic assessment

Initial Management: Upon admission (Day 1), the patient was diagnosed with “schizophrenia” and “pulmonary infection.” Initial treatment included supportive fluid therapy (2000 ml/day), anti-infective therapy with ceftriaxone (2.0 g/day), and mucolytic therapy with ambroxol (60 mg/day). Urinary catheterization was performed due to retention. Given the clinical presentation of catatonia, Modified Electroconvulsive Therapy (MECT) was recommended but refused by the family. Although benzodiazepines are standard treatment for catatonia, they were withheld due to the concurrent pulmonary infection and their potential to suppress respiration and aggravate respiratory symptoms. Risperidone (1 mg/day) was initiated to address psychotic symptoms.

On Day 2, the patient’s condition remained unchanged: mute, immobile, refusing oral intake, and exhibiting waxy flexibility. Attempts at oral hydration resulted in choking and coughing, indicating dysphagia. A nasogastric tube was inserted for nutritional support (1000 ml/day). The risperidone dose was increased to 2 mg/day. MECT was recommended again but was once again declined by the family.

Re-evaluation and Differential Diagnosis: By Day 6, the patient remained mute and immobile. Physical examination revealed generalized hypotonia (flaccid limbs) and hyporeflexia, which was inconsistent with the rigidity typical of catatonic stupor or the resistance seen in negativism. Sensory examination suggested hypoesthesia in both lower limbs; pathological reflexes were negative. Follow-up chest CT showed significant absorption of infectious foci, and inflammatory markers had normalized (WBC 5.2×10^9^/L, CRP 2.1 mg/L). Ceftriaxone and ambroxol were discontinued, and risperidone was titrated to 3 mg/day. The urinary catheter was removed. The chief physician noted the discrepancy between the patient’s flaccid paralysis and typical catatonic rigidity. Combined with the antecedent infection, a peripheral neuropathy was suspected. A lumbar puncture was recommended to rule out organic causes. However, the family strongly refused, attributing the symptoms to a recurrence of schizophrenia similar to his first episode.

Clinical Progression and Confirmed Diagnosis: On Day 17, the patient showed partial improvement: he could sit up with assistance and move his lower limbs horizontally on the bed but remained mute with slow swallowing and easy choking. By Day 20, the patient began to speak, and hallucinations/delusions had largely remitted. He could sit at the bedside but was unable to stand. Notably, he whispered to his mother, “Ask the doctor if my legs will get better?” This subjective complaint of weakness and desire for recovery, combined with persistent dysphagia and history of infection, strongly pointed away from catatonia (which usually resolves alongside psychotic symptoms) and towards Guillain-Barré Syndrome (GBS). Following further persuasion, the family consented to a lumbar puncture. On Day 22, the patient’s speech increased, though his voice remained low. The nasogastric tube was removed as swallowing improved. Upper limb movement was normal, but lower limbs required support for movement. Cerebrospinal Fluid (CSF) Analysis: Routine: WBC 1.2×10^6^/L, RBC 0.01×10^9^/L, Pandy’s test (+) (which involves mixing of CSF with phenol has been a routine examination to detect the presence of globulins in CSF for the past century) (14). Biochemistry: Protein 0.61 g/L (Reference: 0.15–0.45 g/L), demonstrating albuminocytologic dissociation. Autoimmune Panel: Autoimmune encephalitis and anti-ganglioside antibodies were negative. Based on the clinical presentation and CSF findings, a diagnosis of Guillain-Barré Syndrome (GBS) was confirmed. The patient was transferred to a general hospital’s neurology department.

Follow-up and Outcomes: At the general hospital, the patient received Intravenous Immunoglobulin (IVIG) at 25 g/day for 5 days. After 7 days of treatment, his speech improved, choking resolved, and he could walk short distances with assistance. He was discharged to continue rehabilitation. Throughout the follow-up period, the patient maintained adherence to risperidone (4 mg/day) as prophylaxis against psychotic relapse. At 3-month follow-up, Psychiatric symptoms were stable. No dysphagia was reported. He could walk independently but slowly for short distances. At 6-month follow-up, Psychiatric stability was maintained. Mild lower limb weakness persisted. At 1year, all symptoms had resolved. The patient reported no discomfort and had returned to normal work and daily life. The patient’s entire diagnostic and treatment course is summarized in **Table 1**.

**Table 1:** Timeline of the episode of care.

Time	Key events (symptoms/exams)	Diagnostic impression	Management/outcomes
3 years prior	First psychotic episode: auditory hallucinations, persecutory delusions, catatonia	Schizophrenia	Aripiprazole 20 mg/day → improved
1 years prior	Experienced 2 relapses due to medication non-adherence; hallucinations/delusions	Schizophrenia	Condition stable on Risperidone 2 mg/day maintenance
1 month prior	Traveled to Hangzhou for employment; suspected medication discontinuation	—	—
2 weeks prior	Lost contact with family	—	—
3 days prior	Found in chaotic living environment; bedridden; disorganized speech, hallucinations, weakness, low voice	Suspected schizophrenia relapse	—
2 days prior	Complete mutism/immobility, refusal of food/water, inability for self-care	Suspected catatonia/psychosis relapse	Emergency admission
Hospital day 1	Vitals: T 38.6 °C, HR 112 bpm; chest: coarse breath sounds, bilateral lower lobe moist rales; urinary retention (distended bladder). Neuro: flaccid limbs, marked hypotonia. MSE: mutism, bedbound; waxy flexibility. Labs: WBC 14.1×10^9^/L, neutrophils 12.16×10^9^/L, hs-CRP 16.1 mg/L; Chest CT: small bilateral lower-lung infectious lesions.	Diagnosed: schizophrenia + pulmonary infection	IV fluids 2000 mL/day, ceftriaxone 2.0 g/day, ambroxol 60 mg/day; urinary catheterization. Risperidone 1 mg/day started
Day 2	Still mute/immobile; waxy flexibility. Dysphagia suspected (choking/coughing with oral intake)	Catatonia considered	Nasogastric feeding 1000 mL/day; risperidone ↑ to 2 mg/day.
Day 6	Persistent mutism/immobility. Neuro: generalized hypotonia (flaccidity) + hyporeflexia; hypoesthesia in both lower limbs. Infection improved: chest CT absorption; WBC 5.2×10^9^/L, CRP 2.1 mg/L	Concern for peripheral neuropathy rather than typical catatonia	Ceftriaxone/ambroxol stopped; risperidone ↑ to 3 mg/day; urinary catheter removed. Lumbar puncture recommended but family refused
Day 17	Partial motor improvement: could sit up with assistance; move lower limbs horizontally; still mute; persistent dysphagia (slow swallowing, easy choking)	Increasing suspicion of neurologic disorder	Continued supportive care
Day 20	Began speaking; hallucinations/delusions largely remitted; could sit at bedside but unable to stand; asked about leg recovery	Catatonia less likely; GBS strongly suspected	Family agreed to lumbar puncture
Day 22	Speech increased (low voice); swallowing improved → NG tube removed. Upper limbs normal; lower limbs weak (needed support). CSF: WBC 1.2×10^6^/L, RBC 0.01×10^9^/L, Pandy (+); protein 0.61 g/L → albuminocytologic dissociation. Autoimmune encephalitis & anti-ganglioside antibodies negative	Guillain–Barré syndrome confirmed	Transferred to general hospital neurology
Neurology stay	Speech improved; choking resolved; able to walk short distances with assistance	Improving GBS	IVIG 25 g/day × 5 days + rehabilitation
3-month follow-up	Psychiatric symptoms stable; no dysphagia; walking independently but slowly for short distances	Stable schizophrenia; residual GBS weakness improving	Continued risperidone 4 mg/day (prophylaxis)
6-month follow-up	Psychiatric stability maintained; mild lower-limb weakness persisted	Residual weakness	Continued risperidone 4 mg/day
1-year follow-up	Symptoms resolved; no discomfort; returned to normal work and daily life	Recovery	Continued risperidone 4 mg/day

IV fluids, Intravenous fluids; MSE, Mental Status Examination; WBC, white blood cell; RBC, Red Blood Cell; GBS, Guillain–Barré syndrome; IVIG, Intravenous Immunoglobulin.

## 4 Discussion

In this case, the patient’s first episode was dominated by hallucinations and persecutory delusions, followed by catatonia-related manifestations, with recovery after treatment. The current episode resembled the initial course: after discontinuation of medication, he developed a syndrome of hallucinations and delusions, which then rapidly progressed to a cluster of symptoms including complete mutism, immobility, and refusal to eat or drink, prompting presentation for care. Given the acute onset, accompanied by fever and localized pulmonary signs (bilateral lower lobe moist rales), timely imaging was crucial (15). Although a chest X-ray is typically the initial modality for evaluating suspected respiratory infections, the patient’s flaccid weakness precluded cooperation with standard X-ray positioning. Therefore, a chest CT was directly administered rather than as a routine exam; this approach mitigated the risk of missing early or small infectious lesions due to the lower sensitivity of plain radiography. Furthermore, to rule out primary organic brain diseases (e.g., encephalitis or epilepsy), brain MRI and EEG were performed. This is clinically crucial, as approximately two-thirds of catatonia cases attributed to general medical conditions have a primary neurologic etiology (16). Specifically, EEG is key to ruling out nonconvulsive status epilepticus presenting as catatonia (17), while MRI is preferred over computed tomography for detecting inflammation, cytotoxic edema, or space-occupying lesions (15). Because history-taking and the mental status examination were difficult, and because cranial MRI and EEG showed no obvious abnormalities, together with the prior diagnosis, the early inpatient assessment favored a relapse of schizophrenia with catatonic features.

The basic treatment of catatonia emphasizes close monitoring and symptomatic supportive care to prevent serious complications such as pneumonia, urinary tract infection, urinary retention, deep vein thrombosis, pressure ulcers, acute kidney injury, and arrhythmias (18). Regarding pharmacotherapy, the Chinese Guidelines for the Diagnosis and Treatment of Schizophrenia (Second Edition) recommend intravenous sulpiride or oral atypical antipsychotics for patients with catatonia (19); a recent review suggests potential benefit from sulpiride, clozapine, and risperidone, whereas evidence for aripiprazole and olanzapine is inconsistent, and haloperidol and quetiapine are mostly reported as unfavorable (20). In this case, because the patient had responded well to risperidone previously, risperidone was continued. Benzodiazepines (especially lorazepam) are generally regarded as first-line treatment for catatonia (21); however, because this patient had a concomitant pulmonary infection and benzodiazepines were considered potentially capable of suppressing respiration and aggravating respiratory symptoms, they were not used (22). ECT is considered one of the safe and effective treatments for catatonia, with reported response rates of 80%–100% (23), and multiple guidelines also list ECT as a first-line therapy (8, 19, 24). In this case, ECT was recommended repeatedly, but the family refused because of concerns about adverse effects; the subsequent diagnostic clarification also indicated that ECT would not have addressed the underlying neurologic etiology.

When a patient with schizophrenia presents with mutism and immobility, clinicians should differentiate catatonia from medication adverse effects (e.g., excessive sedation), delirium secondary to infection or metabolic abnormalities, neuroleptic malignant syndrome (NMS), and neurological disorders capable of causing muscle weakness or bulbar involvement (e.g., GBS). In this case, several features were atypical for catatonia, including flaccid weakness rather than increased muscle tone, diminished sensation in both lower extremities, dysphagia with choking on water and dysphonia, suggesting possible bulbar or peripheral nerve involvement, and persistent weakness after remission of psychiatric symptoms. Although rare cases of risperidone-induced GBS have been reported in the literature (25), this possibility was considered unlikely in our patient because risperidone had been discontinued prior to admission, and the serum drug level was undetectable (0 ng/mL) upon presentation. Antipsychotic treatment, particularly with relatively potent D2-blocking agents such as risperidone, should be used cautiously because of the potential risk of NMS (26). However, in this case, the major neurological manifestations were already present before risperidone was reintroduced, and psychiatric symptoms improved after risperidone was resumed, whereas neurological deficits improved only after immunotherapy, making risperidone-induced NMS, acute medication toxicity, and excessive sedation clinically unlikely. Although antipsychotic use may increase the risk of aspiration and pneumonia (27), this mechanism appeared less likely in our patient, given the absence of obvious sialorrhea during prior risperidone treatment and collateral evidence of probable medication nonadherence before admission. Although autonomic instability may occur in GBS, catatonia, and NMS, it is not specific and therefore cannot be used in isolation as evidence supporting the diagnosis of GBS. In our patient, flaccid weakness, decreased or absent deep tendon reflexes, sensory disturbance, dysphagia, and subsequent cerebrospinal fluid albuminocytologic dissociation collectively favored peripheral nerve involvement, supporting GBS over catatonia or NMS.

GBS is an immune-mediated acute inflammatory peripheral neuropathy, typically reaching a peak severity at approximately 2 weeks, and is characterized primarily by nerve root and peripheral nerve involvement, with an incidence of approximately (0.30–6.08) per 100,000 population (28). Although accumulating evidence suggests an association between immune-mediated neurologic diseases and mental health (29), and a systematic review indicates that patients with GBS may experience anxiety, depression, acute reactive psychosis, and post-traumatic stress disorder (30), these psychiatric symptoms are mostly related to psychological trauma from intensive care, mechanical ventilation, severe weakness, and functional limitation (31). Existing studies also suggest that some autoimmune neurologic diseases may increase the risk of schizophrenia, but there is no clear association between GBS and schizophrenia (32). In this case, the patient had a clear history of antipsychotic discontinuation before onset, but had also been out of contact for approximately two weeks, making the exact temporal relationship between infection and psychiatric symptoms difficult to determine. Overall, the clinical course was considered more consistent with antipsychotic discontinuation leading to relapse of schizophrenia, reduced self-care capacity, secondary pulmonary infection, and subsequent GBS, although the possibility that infection occurred first and contributed to the psychiatric deterioration cannot be excluded.

The Chinese Guidelines for the Diagnosis and Treatment of GBS (2024) recommend initiating immunotherapy as early as possible to control disease progression and reduce disability, including intravenous immunoglobulin (IVIG) and plasma exchange, which have comparable efficacy, while IVIG is more convenient to implement; routine use of glucocorticoids is not recommended (33). At the same time, attention should be paid to respiratory and swallowing management, nutritional support, ECG monitoring, monitoring and management of complications, and rehabilitation (33). In this case, the severity of GBS was relatively mild; after psychiatric symptoms were controlled, the patient was transferred to a general hospital, where he received IVIG and rehabilitation and ultimately recovered gradually.

This case suggests that, for patients with previously diagnosed mental disorders who are unable to cooperate with examination, clinicians may be prone to anchoring bias—prioritizing a psychiatric explanation—thereby missing comorbid neurologic disease. If the presentation were misjudged as catatonia, lorazepam in the context of infection might aggravate respiratory symptoms and could potentially affect neuromuscular weakness (34), and ECT would lack therapeutic relevance to an underlying neurologic etiology; both could delay appropriate neurologic management. Therefore, when evaluating uncooperative psychiatric patients—especially those with antecedent events such as infection—clinicians should repeatedly perform neurologic examinations and arrange relevant tests as early as possible; lumbar puncture should be considered when necessary to exclude comorbid neurologic disorders such as GBS.

This is a single-case report. Early neurologic assessment was affected by poor cooperation, and a standardized catatonia rating scale (e.g., the Bush–Francis Catatonia Rating Scale, BFCRS) was not used to quantify symptoms. In addition, nerve conduction studies/electromyography, which could have further supported the diagnosis of demyelinating polyneuropathy, were not completed during the admission at our hospital. Nonetheless, in light of the evolution of the clinical course, serial physical examination findings, and CSF albuminocytologic dissociation, the final diagnosis and transfer for treatment remained reasonable.

## Data Availability

The original contributions presented in the study are included in the article/supplementary material. Further inquiries can be directed to the corresponding author.

## References

[1] Working Group on the Third Edition of China’s Guidelines for the Prevention and Treatment of Schizophrenia . Proposal of Chinese clinical practice guidelines for the prevention and treatment of schizophrenia, 3rd edition. Chin J Psychiatry. (2023) 56:331–5. doi:10.3760/cma.j.cn113661-20230520-00104. PMID: 41912385

[2] Kline CL Suzuki T Simmonite M Taylor SF . Catatonia is associated with higher rates of negative affect amongst patients with schizophrenia and schizoaffective disorder. Schizophr Res. (2024) 263:208–13. doi:10.1016/j.schres.2022.09.001. PMID: 36114099

[3] Hirjak D Brandt GA Fritze S Kubera KM Northoff G Wolf RC . Distribution and frequency of clinical criteria and rating scales for diagnosis and assessment of catatonia in different study types. Schizophr Res. (2024) 263:93–8. doi:10.1016/j.schres.2022.12.019. PMID: 36610862

[4] World Health Organization . International classification of diseases 11th revision (ICD-11) (2021). Available online at: https://icd.who.int/browse11 (Accessed April 5, 2026).

[5] American Psychiatric Association . Diagnostic and Statistical Manual of Mental Disorders 5th edn, text revision (DSM-5-TR). Washington, DC: American Psychiatric Association (2022).

[6] Braff DL Ryan J Rissling AJ Carpenter WT . Lack of use in the literature from the last 20 years supports dropping traditional schizophrenia subtypes from DSM-5 and ICD-11. Schizophr Bull. (2013) 39:751–3. doi:10.1093/schbul/sbt068. PMID: 23674819 PMC3686462

[7] Hirjak D Rogers JP Wolf RC Fricchione GL Latorre S Northoff G . Catatonia. Nat Rev Dis Primers. (2024) 10:49. doi:10.1038/s41572-024-00534-w. PMID: 39025858

[8] Rogers JP Oldham MA Fricchione G Northoff G Wilson JE Mann SC . Evidence-based consensus guidelines for the management of catatonia: recommendations from the British Association for Psychopharmacology. J Psychopharmacol. (2023) 37:327–69. doi:10.1177/02698811231158232. PMID: 37039129 PMC10101189

[9] Heckers S Walther S . Catatonia. N Engl J Med. (2023) 389:1797–802. doi:10.1056/NEJMra2116304. PMID: 37937779

[10] Wiegers EJA Jacobs BC . New insights in the immune treatment of Guillain-Barré syndrome. Curr Opin Neurol. (2025) 38:471–7. doi:10.1097/WCO.0000000000001408. PMID: 40748027 PMC12419018

[11] Molloy R Mynes I Pope N . Understanding the experience of diagnostic overshadowing associated with severe mental illness from the consumer and health professional perspective: a qualitative systematic review protocol. JBI Evid Synth. (2021) 19:1362–8. doi:10.11124/JBIES-20-00244. PMID: 33165171

[12] Edinoff AN Kaufman SE Hollier JW Virgen CG Karam CA Malone GW . Catatonia: Clinical overview of the diagnosis, treatment, and clinical challenges. Neurol Int. (2021) 13:570–86. doi:10.3390/neurolint13040057. PMID: 34842777 PMC8628989

[13] Covino M Romozzi M Simeoni B Di Paolantonio A Sabatelli M Franceschi F . Guillain–Barré syndrome from an emergency department view: how to better predict the outcome? Neurol Res. (2022) 44:964–8. doi:10.1080/01616412.2022.2075661. PMID: 35580194

[14] Bullock FN Fleischhacker HH . Interpretation of the pandy test. Acta Psychiatr Scand. (1951) 26:149–53. doi:10.1111/j.1600-0447.1951.TB09665.x. PMID: 14902526

[15] Wilson JE Oldham MA Francis A Perkey D Kramer E Jiang SX . Catatonia: american psychiatric association resource document. J Acad Consultation-Liaison Psychiatry. (2025) 66:277–99. doi:10.1016/j.jaclp.2025.05.001. PMID: 40368005

[16] Hosseini P Whincup R Devan K Ghanem DA Fanshawe JB Saini A . The role of the electroencephalogram (EEG) in determining the aetiology of catatonia: a systematic review and meta-analysis of diagnostic test accuracy. EClinicalMedicine. (2023) 56:101808. doi:10.1016/j.eclinm.2022.101808. PMID: 36636294 PMC9829703

[17] Ogyu K Kurose S Uchida H Kanemoto K Mimura M Takeuchi H . Clinical features of catatonic non-convulsive status epilepticus: a systematic review of cases. J Psychosom Res. (2021) 151:110660. doi:10.1016/j.jpsychores.2021.110660. PMID: 34768095

[18] Connell J Oldham M Pandharipande P Dittus RS Wilson A Mart M . Malignant catatonia: a review for the intensivist. J Intensive Care Med. (2023) 38:137–50. doi:10.1177/08850666221114303. PMID: 35861966

[19] Zhao JP Shi SX . Chinese guidelines for the prevention and treatment of schizophrenia. Beijing: China Medical Electronic Audio and Video Press (2015) p. 105–10.

[20] Redon M Virolle J Montastruc F Taïb S Revet A Da Costa J . The use of antipsychotics in the treatment of catatonia: a systematic review. Eur Psychiatry. (2025) 68:e48. doi:10.1192/j.eurpsy.2025.9. PMID: 40123412 PMC12041727

[21] Caroff SN Ungvari GS Gazdag G . Treatment of schizophrenia with catatonic symptoms: a narrative review. Schizophr Res. (2024) 263:265–74. doi:10.1016/j.schres.2022.11.015. PMID: 36404216

[22] Vozoris NT . Do benzodiazepines contribute to respiratory problems? Expert Rev Respir Med. (2014) 8:661–3. doi:10.1586/17476348.2014.957186. PMID: 25193249

[23] Karl S Sartorius A Aksay SS . Catatonia and ECT across the lifespan. Schizophr Res. (2024) 263:246–51. doi:10.1016/j.schres.2023.04.004. PMID: 37087393

[24] Hasan A Falkai P Wobrock T Lieberman J Glenthoj B Gattaz WF . World Federation of Societies of Biological Psychiatry (WFSBP) guidelines for biological treatment of schizophrenia, part 1: update 2012 on the acute treatment of schizophrenia and the management of treatment resistance. World J Biol Psychiatry. (2012) 13:318–78. doi:10.3109/15622975.2012.696143. PMID: 22834451

[25] Kıcalı GD . Guillain-Barre like syndrome associated with risperidone use: A case report. J Clin Psy. (2022) 25:426–9. doi:10.5505/kpd.2022.70846

[26] Oumary OE Laaraj H Ouhamou M Mouhadi K Doufik J Akebour K . Case Report: Atypical neuroleptic Malignant syndrome induced by paliperidone palmitate—diagnostic challenges and clinical considerations. Front Psychiatry. (2026) 17:1718815. doi:10.3389/fpsyt.2026.1718815. PMID: 41756567 PMC12933270

[27] Sayegh AA Naguy A . Psychotropic-related aspiration hazards: A call for action. Prim Care Companion CNS Disord. (2026) 28:25lr04102. doi:10.4088/PCC.25lr04102. PMID: 41671041

[28] Guo J Pang X . Guillain-barré syndrome. Chin J Neurol. (2023) 56:924–31. doi:10.3760/cma.j.cn113694-20230309-00171. PMID: 41912385

[29] Levison LS Thomsen RW Andersen H . Increased risk of depression after Guillain-Barré syndrome. Muscle Nerve. (2023) 67:497–505. doi:10.1002/mus.27816. PMID: 36906822

[30] Li CMF Wong S Fabiano N Li C McShane M Iansavitchene A . Systematic review: mental health outcomes in Guillain-Barré syndrome and chronic inflammatory demyelinating polyneuropathy. Can J Neurol Sci. (2025), 1–10. doi:10.1017/cjn.2025.10107. PMID: 40485150

[31] Le Guennec L Brisset M Viala K Essardy F Maisonobe T Rohaut B . Post-traumatic stress symptoms in Guillain-Barré syndrome patients after prolonged mechanical ventilation in ICU: a preliminary report. J Peripher Nerv Syst. (2014) 19:218–23. doi:10.1111/jns.12087. PMID: 25403788

[32] Cao Y Ji S Chen Y Zhang X Ding G Tang F . Association between autoimmune diseases of the nervous system and schizophrenia: a systematic review and meta-analysis of cohort studies. Compr Psychiatry. (2023) 122:152370. doi:10.1016/j.comppsych.2023.152370. PMID: 36709559

[33] Chinese Society of Neurology, Peripheral Neuropathy Collaboration Group of Chinese Society of Neurology . Chinese guideline on diagnosis and treatment of Guillain-Barré syndrome 2024. Chin J Neurol. (2024) 57:936–44. doi:10.3760/cma.j.cn113694-20240220-00099. PMID: 41912385

[34] Seldenrijk A Vis R Henstra M Ho Pian K van Grootheest D Salomons T . Systematic review of the side effects of benzodiazepines. Ned Tijdschr Geneeskd. (2017) 161:D1052. 29076441

